# Shenlian (SL) Decoction, a Traditional Chinese Medicine Compound, May Ameliorate Blood Glucose via Mediating the Gut Microbiota in db/db Mice

**DOI:** 10.1155/2022/7802107

**Published:** 2022-02-09

**Authors:** Rui-xi Sun, Wei-jun Huang, Yao Xiao, Dou-dou Wang, Guo-hua Mu, He Nan, Bo-ran Ni, Xiao-qiang Huang, Hsuan-chuan Wang, Yi-fan Liu, Qiang Fu, Jin-xi Zhao

**Affiliations:** ^1^Beijing University of Chinese Medicine, Beijing, China; ^2^Key Laboratory of Chinese Internal Medicine of Ministry of Education and Beijing, Dongzhimen Hospital Affiliated to Beijing University of Chinese Medicine, Beijing University of Chinese Medicine, Beijing, China; ^3^Nephropathy Department, Beijing University of Chinese Medicine Third Affiliated Hospital, Beijing University of Chinese Medicine, Beijing, China; ^4^Section II of Endocrinology & Nephropathy Department, Dongzhimen Hospital Affiliated to Beijing University of Chinese Medicine, Beijing University of Chinese Medicine, Beijing, China

## Abstract

Shenlian (SL) decoction is a herbal formula composed of Coptis and ginseng, of which berberine and ginsenoside are the main constituents. Even though SL decoction is widely used in treating diabetes in China, the mechanism of its antidiabetes function still needs further study. Gut microbiota disorder is one of the important factors that cause diabetes. To explore the effect of SL decoction on intestinal microbiota, gut microbiota of mice was analyzed by sequencing the gut bacterial 16S rRNA V3+V4 region and metagenomics. In this study, results demonstrated that SL decoction had a better hypoglycemic effect and *β* cell protection effect than either ginseng or Coptis chinensis. Alpha diversity analysis showed that all interventions with ginseng, Coptis, and SL decoction could reverse the increased diversity and richness of gut microbiota in db/db mice. PCoA analysis showed oral SL decoction significantly alters gut microbiota composition in db/db mice. 395 OTUs showed significant differences after SL treatment, of which 37 OTUs enriched by SL decoction showed a significant negative correlation with FBG, and 204 OTUs decreased by SL decoction showed a significant positive correlation with FBG. Results of KEGG analysis and metagenomic sequencing showed that SL decoction could reduce the *Prevotellaceae*, *Rikenellaceae*, and *Helicobacteraceae*, which were related to lipopolysaccharide biosynthesis, riboflavin metabolism, and peroxisome, respectively. It could also upregulate the abundance of *Bacteroidaceae*, which contributed to the metabolism of starch and sucrose as well as pentose-glucuronate interconversions. In the species level, SL decoction significantly upregulates the relative abundance of Bacteroides_acidifaciens which showed a significant negative correlation with FBG and was reported to be a potential agent for modulating metabolic disorders such as diabetes and obesity. In conclusion, SL decoction was effective in hypoglycemia and its mechanism may be related to regulating gut microbiota via upregulating Bacteroides_acidifaciens.

## 1. Introduction

Diabetes is a chronic glycemic metabolism disorder disease, which is associated with excessive calorie intake, lack of exercise, genetic predisposition, etc. According to the data from International Diabetes Federation (IDF), there are 463 million people that have been diagnosed with diabetes worldwide in 2019 and the number is expected to increase to 700 million by 2045 [1]. Along with the increasing prevalence of diabetes, its complications are also becoming big threats to human health. Increasing attention has been focused on diabetes. Mechanism researches have made great progress, and many effective drugs for diabetes have been developed by now. Except for modern medicines, Chinese herbal medicine has been proved to be a good alternative therapy for diabetes. For example, a study from Gao et al. demonstrated that TCM intervention could reduce the conversion rate of IGT to diabetes and improve insulin resistance [2]. Another randomized clinical trial showed that JinQi Jiangtang tablets (a Chinese patent medicine) could effectively reduce the incidence of diabetes mellitus and even reverse the prediabetes to normal [3, 4]. Tianqi capsule, another Chinese patent medicine, also has been proved to decrease the incidence of T2DM in subjects with IGT. Therefore, traditional Chinese medicine is worthy to be further studied.

Shenlian decoction, which consists of Coptis and ginseng, is widely used in treating diabetes in China. Using Coptis and ginseng in treating diabetes can be traced back to the Tang dynasty, which was recorded in the book Qianjin fang, written by Sun Simiao. In the Ming dynasty, these two herbs were firstly used as a prescription and recorded as Shenlian decoction in the book Wanbinghuichun. Nowadays, many studies showed that both Coptis and ginseng have the function of mediating glucose metabolism [5–9]. Besides, a study from Yuan et al. demonstrated the function of Shenlian decoction in protecting pancreatic *β* cell [10]. However, further studies are needed to clarify the mechanism of its antidiabetes function. In this study, we try to uncover the mechanism from the point of mediating the gut microbiota.

## 2. Materials and Methods

### 2.1. Drugs and Reagents

Coptis chinensis and Panax ginseng were purchased from the Beijing Kangmei Pharmaceutical Co. LTD, and the contents of representative chemical compositions were determined by the Research and Experimental Center of Beijing University of Chinese Medicine (Table 1). Metformin hydrochloride tablets were purchased from the Sino-American Shanghai Squibb Pharmaceuticals Ltd.

### 2.2. Animal Treatment and Fecal Sample Collection

All animal experiments were performed with the approval of the Ethics Committee for Experimental Animals of the Institute of Basic Theory for Chinese Medicine. Six-week-old male C57BL/KsJ-db/db mice and their normal littermates (db/m) were purchased from Changzhou Cavens Experimental Animal Co. LTD. (Approval Number: SCXK (su) 2016-0010). All animals were housed in an air-conditioned animal experiment room with a stable temperature of 22–24°C, humidity of 50–60%, a 12-hour light and dark cycle, and free access to water and food (standard chow diet). The db/db mice with FBG levels above 13.9 mmol/l were randomly allocated into 5 groups (*n* = 8), including the diabetic control (DC) group (distilled water, 0.2 ml/10 g), Huanglian (HL) group (Coptis chinensis, 4.55 g/kg), Rensheng (RS) group (Panax ginseng, 0.455 g/kg), Shenlian (SL) group (Coptis chinensis and Panax ginseng, 4.55 g and 0.455 g/kg, respectively), and metformin (Met) group (metformin, 0.228 g/kg). The db/m mice were used as normal control (NC group). Interventions were conducted by gavage (0.2 ml/10 g) for 8 weeks. Fasting blood glucose (FBG) was measured using a blood glucose meter (Johnson & Johnson) in the tail vein blood once 4 weeks, for 8 weeks. After 8 weeks, fresh fecal samples from each animal were collected via a clean catch method, in which pellets are expressed directly into a sterile centrifuge tube and stored at -80°C. Fecal samples were sent to Shanghai Majorbio Bio-Pharm Technology Co., Ltd., for further processing.

### 2.3. Immunohistochemistry

The process of immunohistochemistry was conducted as previously [11]. The antibody of insulin was purchased from Proteintech (catalog: 15848-1-AP).

### 2.4. DNA Extraction, Library Construction, and Metagenomic Sequencing

Total genomic DNA was extracted from feces samples using the E.Z.N.A.® Soil DNA Kit (Omega Bio-Tek, Norcross, GA, U.S.) according to the manufacturer's instructions. The process of library construction and metagenomic sequencing was conducted as previously [12].

### 2.5. Sequence Quality Control and Genome Assembly

The data were analyzed on the free online platform of the Majorbio Cloud Platform (http://www.majorbio.com). The paired-end Illumina reads were trimmed for adaptors and low-quality reads (length < 50 bp or with a quality value < 20 or having *N* bases) using fastp [13]. Reads were aligned to the mouse genome by BWA (http://bio-bwa.sourceforge.net, version 0.7.9a), and any hit associated with the reads and their mated reads were removed. Metagenomic data were assembled using MEGAHIT [14, 15] (https://github.com/voutcn/megahit, version 1.1.2). Contigs with the length being or over 300 bp were selected as the final assembling result, and then, the contigs were used for further gene prediction and annotation.

### 2.6. Species Annotation and Evaluation

Sample sequencing results were clustered. All sequences were divided into OTUs (Operational Taxonomic Units) according to different similarity levels using Uparse. The representative sequences of OTUs with their relative abundance were used to calculate community richness, community diversity, and community coverage index by mothur (version v.1.30.1). Then, the relative abundance table of representative sequences of OTUs was used for UniFrac principal coordinate analysis (PCoA) (Lozupone and Knight, 2005), species difference analysis, redundancy analysis, and correlation heatmap analysis.

### 2.7. Gene Prediction, Taxonomy, and Functional Annotation

Open reading frames (ORFs) from each assembled contig were predicted using MetaGene [16] (http://metagene.cb.k.u-tokyo.ac.jp/). The predicted ORFs (no less than 100 bp) were retrieved and translated into amino acid sequences with NCBI translation table. A nonredundant gene catalog was constructed by CD-HIT [17] (http://www.bioinformatics.org/cd-hit/, version 4.6.1) with 90% sequence identity and 90% coverage. Reads after quality control were mapped to nonredundant gene catalog with 95% identity using SOAP aligner [18] (http://soap.genomics.org.cn/, version 2.21), and gene abundance in each sample was evaluated. Representative sequences of nonredundant gene catalog were aligned to NCBI NR database with *e* value cutoff of 1*e* − 5 using Diamond [19]. The KEGG annotation was conducted with Diamond (http://www.diamondsearch.org/index.php, version 0.8.35) against the Kyoto Encyclopedia of Genes and Genomes database (http://www.genome.jp/keeg/) with an *e* value cutoff of 1*e* − 5.

### 2.8. Statistical Analysis

The data are expressed as mean ± SEM and analyzed using SPSS 22.0. Significant differences between the two groups were evaluated by Student's *t*-test or Welch's *t*-test for samples that were normally distributed, and the Wilcoxon rank-sum test for samples that were not normally distributed. Significant differences among three or more groups were evaluated by one-way ANOVA with Bonferroni's multiple comparison test. The level of significance was set at *P* < 0.05; ^∗^*P* < 0.05; ^∗∗^*P* < 0.01; ^∗∗∗^*P* < 0.001.

## 3. Results

### 3.1. Fasting Blood Glucose, Body Weight, and HOMA-IR Immunohistochemistry of db/db Mice

**Figure 1:**
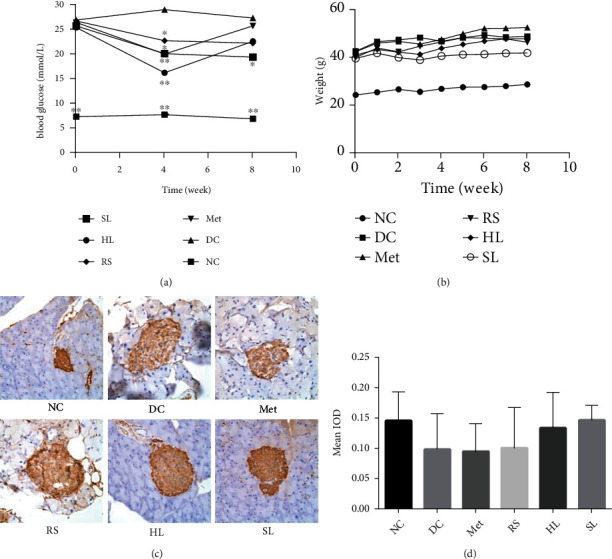
Effects on (a) FBG, (b) body weight, and (c, d) HOMA-IR. Immunohistochemistry of insulin in islet tissue. ^∗^*P* < 0.05 vs. DC group; ^∗∗^*P* < 0.01; ^∗∗∗^*P* < 0.001.

FBG and body weight were evaluated after 6 h of food removal in the morning [20]. As shown in Figure 1(a), during 8 weeks of observation, db/db mice presented significant hyperglycemia compared with the wild-type controls (*P* < 0.001). Metformin, HL, RS, and SL decoction significantly lowered blood glucose in the first 4 weeks (*P* < 0.01, 0.05, 0.01, and 0.05). However, in the eighth week, only the SL group had significantly lower blood glucose levels than the diabetic control group (*P* < 0.05). As for body weight, no significant difference was observed in the HL group, RS group, SL group, and Met group, compared with the DC group (Figure 1(b)). Immunohistochemistry of insulin in islet tissue was conducted to evaluate the function of *β* cells. Results showed that less insulin was detected in db/db mice compared with db/m mice (*P* = 0.059), and HL (*P* = 0.278) and SL decoction (*P* = 0.053) could improve the insulin generation in islet tissue. The efficacy of SL decoction seems to be better than either RS or HL in promoting the insulin generation (Figures 1(c) and 1(d)).

### 3.2. Alpha Diversity Analysis of Bacterial Relative Abundances

**Figure 2:**
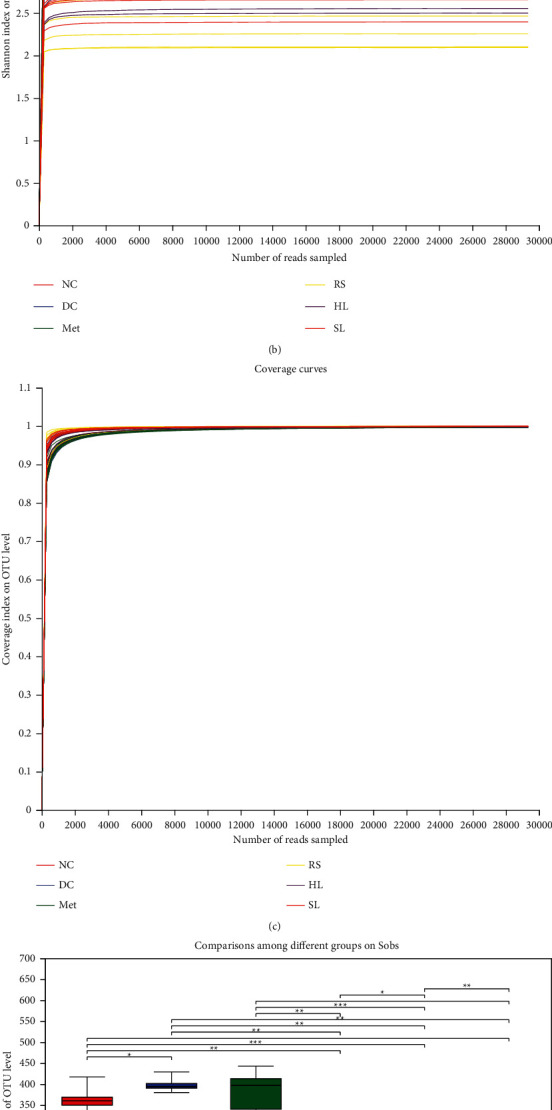
Alpha diversity analysis of bacterial relative abundances. (a) Sobs, (b) Shannon, and (c) coverage diversity curves of gut microbiota community, which reflect the richness, diversity, and coverage of the community, respectively. Comparisons among different groups on (d) Sobs and (e) Shannon of gut microbiota were significant. ^∗^*P* < 0.05; ^∗∗^*P* < 0.01.

To explore the effect of SL decoction on intestinal microbiota, gut microbiota of mice was analyzed by sequencing the gut bacterial 16S rRNA V3+V4 region and metagenomics. First, we used MiSeq run to analyze the structural changes of gut microbiota. In total, 2176480 usable raw sequences and 3222 OTUs were obtained, and a nonredundant catalog of 1609093 genes was constructed. Coverage (community coverage) diversity curves indicated that the sequencing results covered almost all the sequences in the sample, which means that the results could reflect the true situation of the microbiota community in the sample (Figures 2(a)–2(c)).

Sobs (the observed richness) and Shannon (the Shannon diversity index) diversity curves revealed that most of the diversity and richness of gut microbiota community had already been captured. The DC group had the significant higher microbiota diversity and richness compared with the NC group (*P* < 0.05). After 8-week treatment, the increased microbiota diversity and richness were successfully reversed by HL, RS, and SL decoction (Figures 2(d) and 2(e)).

### 3.3. Composition Changes of Gut Microbiota

**Figure 3:**
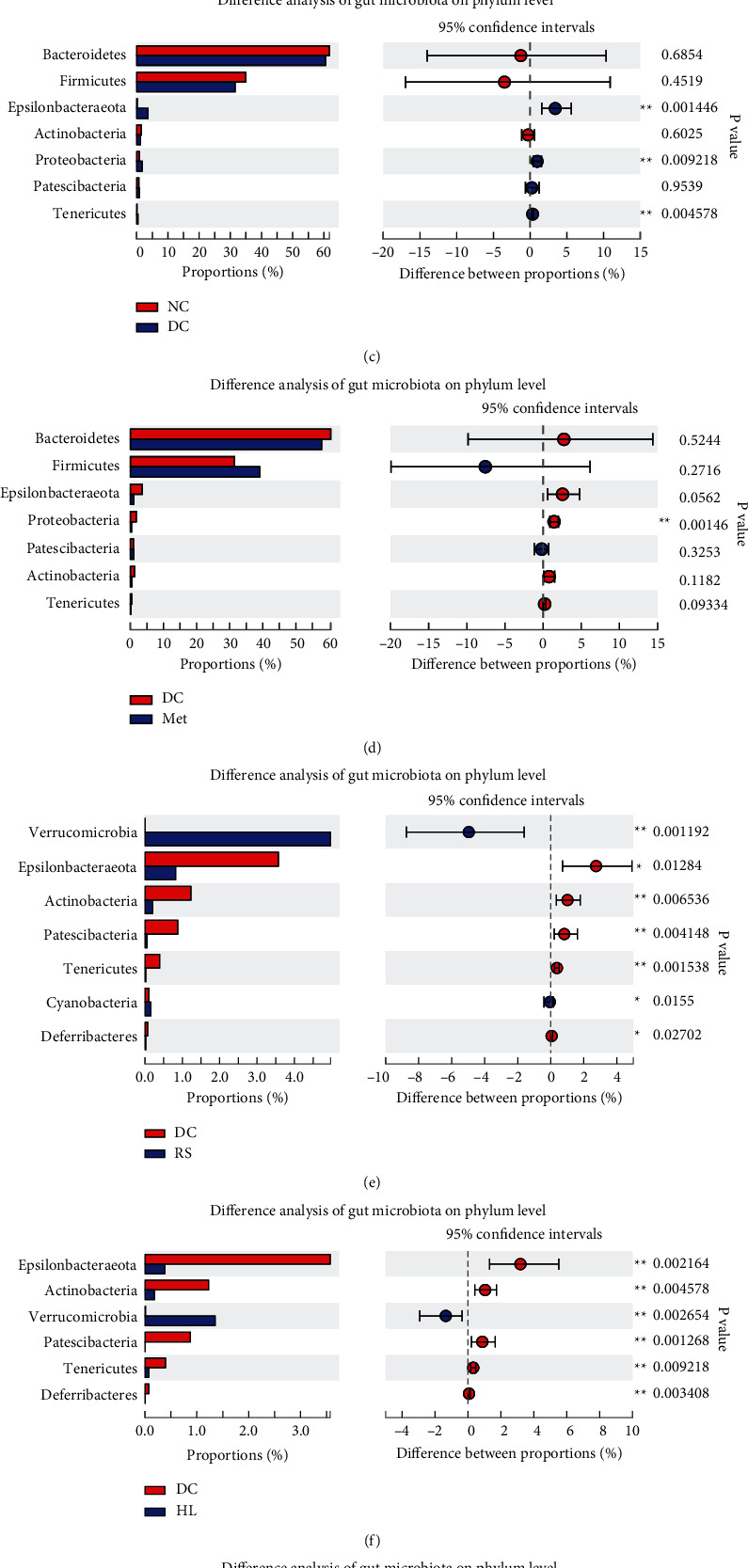
(a) Principal coordinate analysis (PCoA) of gut microbiota for mice. Weighted UniFrac PCoA plot and based on OTU abundance, each point represents the placenta microbiota of a sample, with main principal component (PC) scores: PC1 = 33.52% and PC2 = 13.26%. (b) The boxplot represents the dispersion of distribution of different groups of samples on PC1. Relative abundance of phylum level was significantly different among (c) NC, (d) Met, (e) RS, (f) HL, (g) SL, and DC groups, ^∗^*P* < 0.05, ^∗∗^*P* < 0.01, compared with the DC group.

PCoA analysis showed a clear separation of the gut microbiota structure between DC and other groups (Figure 3(a)), especially when compared to the SL group with the DC group (Figure 3(b)). Further, the relative levels of gut microbiota at the phylum level were compared among groups. In the DC group, the abundances of Epsilonbacteraeota, Proteobacteria, and Tenericutes were significantly higher than the NC group (Figure 3(c)). Notably, HL, RS, and SL decoction could decrease the abundance of Epsilonbacteraeota and Tenericutes, and metformin decreased Proteobacteria abundance. What is more, HL, RS, and SL also increased the abundance of Verrucomicrobia and decreased the abundance of Actinobacteria, Patescibacteria, and Deferribacteres (Figures 3(d)–3(g)).

### 3.4. Correlation between Gut Microbiota Structure and FBG

**Figure 4:**
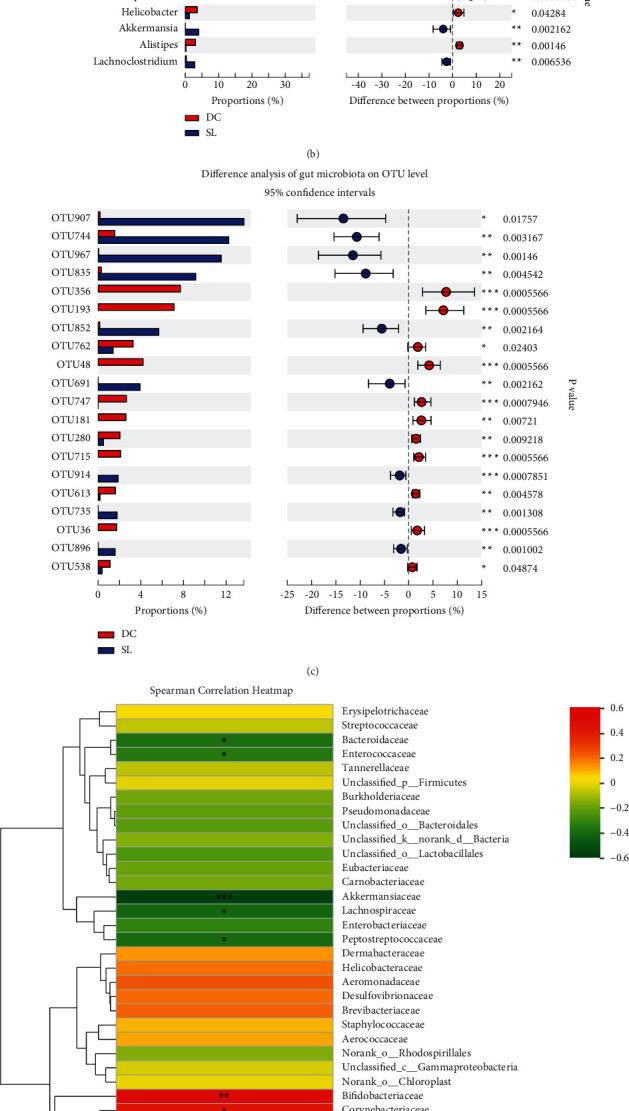
Significantly different between DC and SL groups of gut microbial community at the (a) family level, (b) genus level, and (c) OTU level. ^∗^*P* < 0.05 and ^∗∗^*P* < 0.01, compared with the DC group. (d) Heatmap analysis indicating Spearman correlation between gut microbiota community and FBG on the family level, correlation *R* values, and *P* values were obtained by calculation; ^∗^*P* < 0.05 and ^∗∗^*P* < 0.01; *R* values were shown in different colors in the diagram.

SL decoction alters gut microbiota composition significantly in db/db mice, the relative levels of gut microbiota were significantly different between DC and SL groups at the family level and genus level (Figures 4(a) and 4(b)), and top 20 OTU differences are shown in Figure 4(c). In order to identify specific gut bacteria that potentially are associated with FBG after SL treatment, Spearman's correlation analysis was performed between the gut microbiota community and FBG (Figure 4(d)). In total, 395 OTUs showed significant differences after SL treatment. 316 OTUs were decreased, and 79 were increased. Among the 79 OTUs enriched by SL, 37 OTUs showed a significant negative correlation with FBG (Supplementary Table 1). These OTUs belonged to Akkermansiaceae (*n* = 2), unclassified_o__Bacteroidales (*n* = 6), unclassified_k__norank_d__Bacteria (*n* = 1), Tannerellaceae (*n* = 2), Ruminococcaceae (*n* = 2), Rikenellaceae (*n* = 1), Peptostreptococcaceae (*n* = 1), norank_o__Rhodospirillales (*n* = 1), Muribaculaceae (*n* = 5), Lachnospiraceae (*n* = 8), Erysipelotrichaceae (*n* = 1), Enterobacteriaceae (*n* = 1), and Bacteroidaceae (*n* = 6) on the family level. Among the 316 OTUs decreased by SL, 204 OTUs showed a significant positive correlation with FBG (Supplementary Table 2); these OTUs belonged to unclassified_o__Bacteroidales (*n* = 6), Tannerellaceae (*n* = 3), Streptococcaceae (*n* = 1), Saccharimonadaceae (*n* = 1), Ruminococcaceae (*n* = 34), Rikenellaceae (*n* = 11), Prevotellaceae (*n* = 9), Peptococcaceae (*n* = 2), norank_o__Gastranaerophilales (*n* = 1), Muribaculaceae (*n* = 66), Moraxellaceae (*n* = 1), Marinifilaceae (*n* = 3), Lactobacillaceae (*n* = 2), Lachnospiraceae (*n* = 36), Helicobacteraceae (*n* = 1), Erysipelotrichaceae (*n* = 5), Eggerthellaceae (*n* = 7), Desulfovibrionaceae (*n* = 1), Deferribacteraceae (*n* = 1), Clostridiales_vadinBB60_group (*n* = 6), Clostridiaceae_1 (*n* = 1), Christensenellaceae (*n* = 2), Burkholderiaceae (*n* = 1), Bifidobacteriaceae (*n* = 1), and Bacteroidaceae (*n* = 2) on the family level.

### 3.5. Changes in Metabolic and Synthetic Functions of Gut Microbiota Caused by SL Decoction

**Figure 5:**
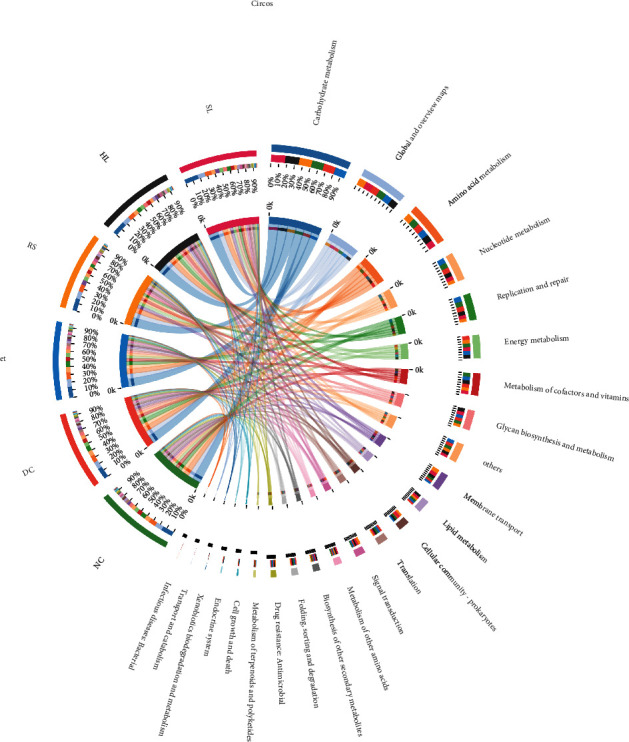
Circos diagram of the KEGG pathways enrichment analysis, the left half circle represents the function abundance composition of the group, and the right half circle represents the distribution ratio of the function.

**Figure 6:**
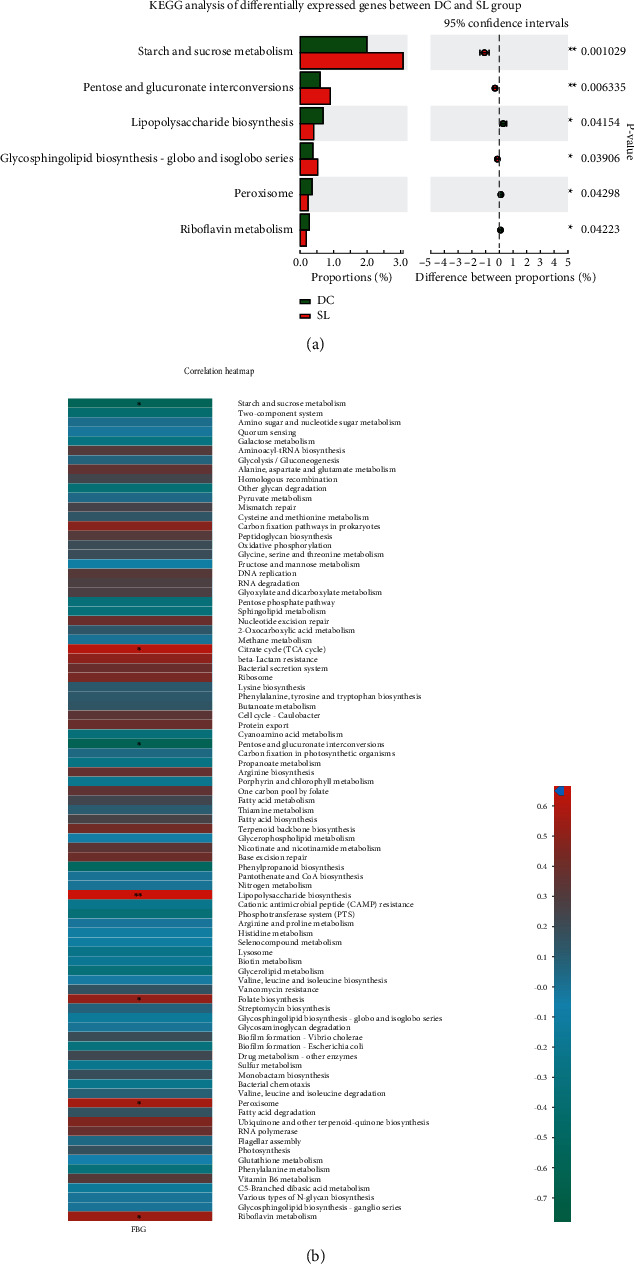
(a) KEGG analysis of differentially expressed genes between DC and SL groups. ^∗^*P* < 0.05 and ^∗∗^*P* < 0.01, compared with the DC group. (b) Correlation heatmap between KEGG analysis and FBG in db/db mice, the shades of color represent the richness of the function; correlation *R* values and *P* values were obtained by calculation; ^∗^*P* < 0.05 and ^∗∗^*P* < 0.01; *R* values were shown in different colors in the diagram.

To investigate the changes in metabolic and synthetic functions within the gut microbiota regulated by SL, the KEGG pathway enrichment analysis was performed. The most enriched functions included carbohydrate metabolism, amino acid metabolism, nucleotide metabolism, replication and repair energy metabolism, metabolism of cofactors and vitamins, and glycan biosynthesis (Figure 5). The KEGG analysis of differentially expressed genes between DC and SL is shown in Figure 6(a), and Spearman correlation between KEGG analysis and FBG is shown in Figure 6(b) on pathway level 3. The abundances of starch and sucrose metabolism, as well as pentose-glucuronate interconversions, showed a significant negative correlation with FBG and significantly increased after the SL treatment. In addition, the relative abundances of riboflavin metabolism, peroxisome, and lipopolysaccharide biosynthesis showed a significant positive correlation with FBG and significantly decreased after the SL treatment.

**Figure 7:**
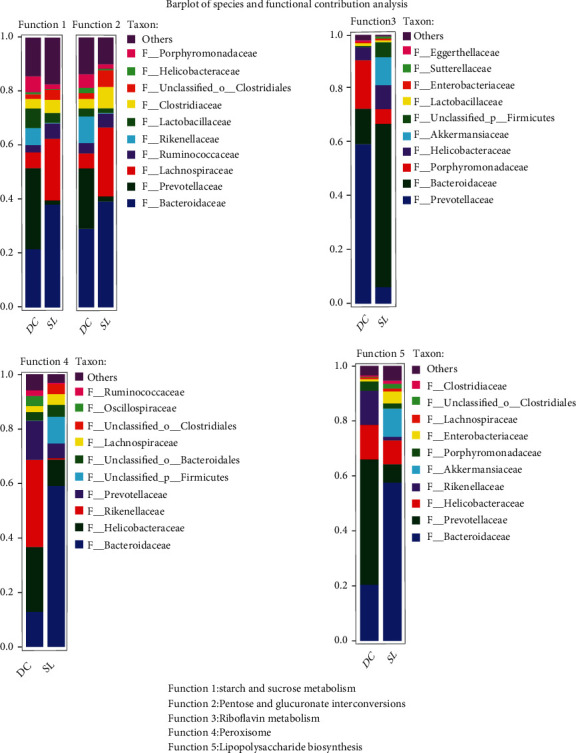
Barplot of species and functional contribution analysis indicates the dominant species composition of a particular function.

**Figure 8:**
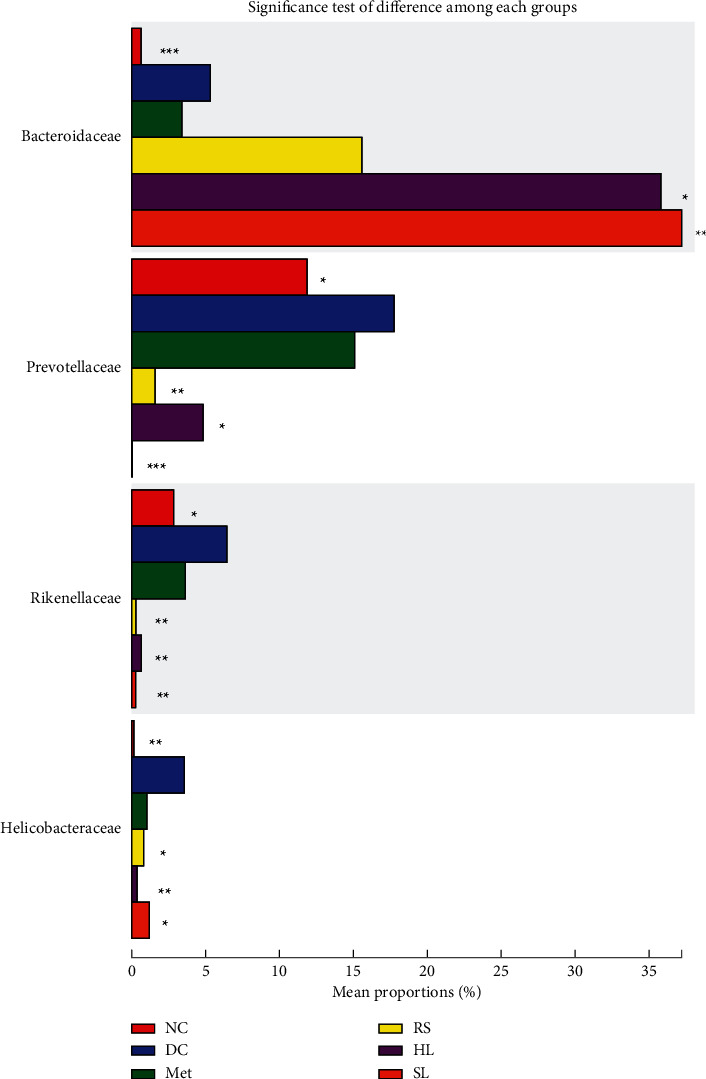
Significance test of difference of Bacteroidaceae, Prevotellaceae, Rikenellaceae, and Helicobacteraceae among each group, ^∗^*P* < 0.05; ^∗∗^*P* < 0.01; ^∗∗∗^*P* < 0.001, compared with the DC group.

With the analysis of contribution capacity of different strains by metagenomic sequencing, Bacteroidaceae was found to display the greatest contribution to starch and sucrose metabolism and pentose and glucuronate interconversions. Prevotellaceae contributed the most to riboflavin metabolism and lipopolysaccharide biosynthesis. As for peroxisome, Rikenellaceae, Helicobacteraceae, and Prevotellaceae did a great contribution (Figure 7). Importantly, SL decoction could significantly decrease the abundance of Prevotellaceae, Rikenellaceae, and Helicobacteraceae and increase the abundance of Bacteroidaceae. The changes in relative abundance on Bacteroidaceae and Prevotellaceae were particularly significant in the SL group, when compared with other treatment groups (Figure 8). What is more, SL decoction could reduce the relative abundance of 21 species positively correlated with FBG (Table 2) belonging to Prevotellaceae, Rikenellaceae, and Helicobacteraceae significantly, which were related to lipopolysaccharide biosynthesis, riboflavin metabolism, and peroxisome. It could also upregulate the relative abundance of 6 species negatively correlated with FBG (Table 3) belonging to Bacteroidaceae, which contributed to the metabolism of starch and sucrose as well as pentose-glucuronate interconversions.

Data of DC and SL groups were shown as relative abundance (%) of species and family in each group. Statistical analysis was performed by the Mann-Whitney *U* test adjusted for multiple testing.

## 4. Discussion

Diabetes is a chronic metabolic disease with high mortality and morbidity, which can lead to multiple organ injuries, including kidney, eyes, heart, nerve, and blood circulation.

Traditional Chinese medicine (TCM) has a long history of treating diabetes, which can trace back to the Han dynasty. During these thousands of years, ancient Chinese doctors accumulated lots of experience in treating diabetes. Shenlian decoction is one of them. An increasing number of studies showed that hypoglycemic Chinese medicines regulate glucose via mediating the gut microbiota. Quan et al. proved that ginseng extract could regulate the microbiota-lcFa-Bat axis, which played an important role in obesity [21]. Berberine, which is the main extract of Coptis chinensis, has been used in treating diabetes, and regulating gastrointestinal microbiota was proved to be the main mechanism of its antidiabetes function [22]. In this study, results demonstrated that Shenlian decoction had a good hypoglycemic function, which was better than either Coptis chinensis or ginseng after 8-week intervention. Results of insulin IHC also indicated that SL decoction had a better effect in protecting the function of *β* cells than either ginseng or Coptis chinensis. Because in the theory of Chinese medicine, the basic mechanism of diabetes is “heat injures qi and yin.” It means that pathogen heat, qi deficiency, and yin deficiency are main syndromes in diabetes patients. Therefore, in the clinic practice, TCM doctors always use Coptis chinensis to clear the heat and together with ginseng to tonify qi and yin. Some studies reported that ginseng failed to decrease blood glucose [23]. The reason is that ginseng can tonify qi and yin but cannot clear pathogen heat. So when using ginseng alone to treat diabetes, the efficacy cannot be good enough.

Increasingly, studies have shown that diabetes has a close relationship with the disorder of gut microbiota [24]. Dysbiosis of gut microbiota can disrupt the intestinal barrier, causing pathogens, proinflammatory cytokines, and harmful metabolites to enter the blood circulation, which contribute to the chronic low-grade inflammation [25]. Dysbiosis of gut microbiota in diabetes also manifests as a decline of short-chain fatty acid (SCFS) production. Low-grade SCFS induced decreased peptide YY and glucagon-like peptide- (GLP-) 1 secretion, increased food intake, and disrupted intestinal barrier [26]. What is more, branched-chain amino acid (BCAA) biosynthesis is promoted when dysbiosis of gut microbiota happens and high BCAA level can cause insulin resistance [27, 28]. In this study, results showed that SL decoction could alter gut microbiota composition in db/db mice. Among the altered OTUs, 37 OTUs showed a significant negative correlation with FBG and 204 OTUs showed a significant positive correlation with FBG. What is more, as with the composition, the function of gut microbiota was also regulated by SL decoction, including starch and sucrose metabolism, pentose-glucuronate interconversions, riboflavin metabolism, peroxisome, and lipopolysaccharide biosynthesis, which were related to the FBS. Further analysis showed that SL decoction could decrease the relative abundance of Prevotellaceae, Rikenellaceae, and Helicobacteraceae and increase the abundance of Bacteroidaceae. These four families contributed mostly to FBS-related functions.

In the species level, SL decoction upregulates the relative abundance of Bacteroides_acidifaciens significantly, which showed a significant negative correlation with FBG and was reported to be a potential agent for modulating metabolic disorders such as diabetes and obesity. As reported, wild-type C57BL/6 mice fed with Bacteroides_acidifaciens were significantly more likely to gain less weight and fat and showed elevated insulin levels in serum, accompanied by increased serum glucagon-like peptide-1 and decreased intestinal dipeptidyl peptidase-4 [29]. Therefore, Bacteroides_acidifaciens upregulation may be the key mechanism for hypoglycemic function of SL decoction. Additionally, results of alpha diversity analysis showed that both HL and SL decoction reduced the diversity and richness of microbiota, which consisted with former studies [30, 31]. This result might mainly be caused by the antibacterial effects of Coptis chinensis [32]. The reduced diversity and richness of gut microbiota in db/db mice could give the beneficial bacteria more space to grow. It means that SL decoction regulates gut microbiota through a “restart microbiota” mode.

In conclusion, SL decoction was proved to be effective in hypoglycemia via regulating gut microbiota, especially the species of Bacteroides_acidifacien. In the future, a RCT should be conducted to prove its efficacy and further mechanism studies should focus on the families of Prevotellaceae, Rikenellaceae, Helicobacteraceae, and Bacteroidaceae.

## Figures and Tables

**Table 1:** Contents of the main identified compounds in SL extract.

Herbs	Compounds	Contents
Coptis chinensis	Berberine	5.80%
	Epiberberine	0.88%
	Coptisine	1.62%
	Palmatine	1.37%

Panax ginseng	Ginsenoside Rg1	0.24%
	Ginsenoside Rb1	0.37%
	Ginsenoside Re	0.27%

**Table 2:** The relative abundance of 21 species reduced by SL.

Family	Species	DC (%)	SL (%)	*P* value
Rikenellaceae	Uncultured_bacterium_g__Rikenellaceae_RC9_gut_group	1.291	0.089	0.0022
Helicobacteraceae	Unclassified_g__Helicobacter	2.647	0.002	0.0008
Prevotellaceae	Uncultured_Bacteroidales_bacterium_g__Prevotellaceae_UCG-001	2.101	0.000	0.0006
Prevotellaceae	Uncultured_Bacteroidales_bacterium_g__Prevotellaceae_UCG-001	0.177	0.000	0.0022
Prevotellaceae	Uncultured_Bacteroidales_bacterium_g__Prevotellaceae_UCG-001	0.128	0.000	0.0022
Rikenellaceae	Uncultured_bacterium_g__Rikenella	0.321	0.000	0.0006
Rikenellaceae	Uncultured_bacterium_g__Alistipes	0.131	0.002	0.0023
Rikenellaceae	Uncultured_bacterium_g__Alistipes	0.224	0.003	0.0157
Prevotellaceae	Unclassified_f__Prevotellaceae	0.013	0.000	0.0213
Rikenellaceae	Uncultured_bacterium_g__Alistipes	0.304	0.000	0.0006
Rikenellaceae	Uncultured_bacterium_g__Alistipes	0.475	0.000	0.0006
Prevotellaceae	Unclassified_f__Prevotellaceae	0.009	0.000	0.0071
Rikenellaceae	Uncultured_bacterium_g__Alistipes	0.064	0.000	0.0006
Rikenellaceae	Unclassified_g__Rikenellaceae_RC9_gut_group	0.007	0.000	0.0019
Rikenellaceae	Unclassified_g__Rikenellaceae_RC9_gut_group	1.745	0.000	0.0006
Prevotellaceae	Gut_metagenome_g__Alloprevotella	7.701	0.000	0.0006
Rikenellaceae	Unclassified_g__Alistipes	0.019	0.000	0.0071
Prevotellaceae	Uncultured_Bacteroidales_bacterium_g__Prevotellaceae_UCG-001	7.136	0.000	0.0006
Rikenellaceae	Unclassified_g__Alistipes	1.329	0.000	0.0006
Prevotellaceae	Unclassified_f__Prevotellaceae	0.006	0.000	0.0211
Prevotellaceae	Unclassified_f__Prevotellaceae	0.255	0.000	0.0072

**Table 3:** The relative abundance of 6 species increased by SL decoction.

Family	Species	DC (%)	SL (%)	*P* value
Bacteroidaceae	Unclassified_g__Bacteroides	0	0.01576	0.002484
Bacteroidaceae	Unclassified_g__Bacteroides	0	0.009372	0.002537
Bacteroidaceae	Unclassified_g__Bacteroides	0	0.1793	0.00259
Bacteroidaceae	Bacteroides_caecimuris	0.4133	1.079	0.02403
Bacteroidaceae	Unclassified_g__Bacteroides	0.004382	0.9819	0.001321
Bacteroidaceae	Bacteroides_acidifaciens	1.55	12.25	0.003167

## Data Availability

The data sets used and/or analyzed during the current study are available from the corresponding author on reasonable request.

## Supplementary Materials

Supplementary 1 Supplementary Table 1: species classification information of 37 OTUs.

Supplementary 2 Supplementary Table 2: species classification information of 204 OTUs.
