# Supplementary material for: Shenlian (SL) Decoction, a Traditional Chinese Medicine Compound, May Ameliorate Blood Glucose via Mediating the Gut Microbiota in db/db Mice
Source: J Diabetes Res. 2022 Feb 9;2022:7802107. doi: 10.1155/2022/7802107 (PMC8855168; doi:10.1155/2022/7802107)
Supplement: Supplementary 1 — Supplementary Table 1: species classification information of 37 OTUs. [file 7802107.f1.pdf]

Phylum	Family	Genus	Species	OTU
Firmicutes	Lachnospiraceae	Lachnoclostridium	[Clostridium]_aldenense	OTU854
Firmicutes	Ruminococcaceae	GCA-900066225	unclassified_g__GCA-900066225	OTU928
Bacteroidetes	unclassified_o__Bacteroidales	unclassified_o__Bacteroidales	unclassified_o__Bacteroidales	OTU695
unclassified_k__norank_d__Bacteria	unclassified_k__norank_d__Bacteria	unclassified_k__norank_d__Bacteria	unclassified_k__norank_d__Bacteria	OTU856
Bacteroidetes	Muribaculaceae	norank_f__Muribaculaceae	uncultured_bacterium_g__norank_f__Muribaculaceae	OTU925
Bacteroidetes	Muribaculaceae	norank_f__Muribaculaceae	uncultured_bacterium_g__norank_f__Muribaculaceae	OTU920
Firmicutes	Lachnospiraceae	Lachnoclostridium	unclassified_g__Lachnoclostridium	OTU810
Firmicutes	Lachnospiraceae	Hungatella	uncultured_bacterium_g__Hungatella	OTU735
Bacteroidetes	Rikenellaceae	Alistipes	unclassified_g__Alistipes	OTU837
Bacteroidetes	Tannerellaceae	Parabacteroides	unclassified_g__Parabacteroides	OTU765
Bacteroidetes	unclassified_o__Bacteroidales	unclassified_o__Bacteroidales	unclassified_o__Bacteroidales	OTU960
Bacteroidetes	Bacteroidaceae	Bacteroides	unclassified_g__Bacteroides	OTU924
Bacteroidetes	unclassified_o__Bacteroidales	unclassified_o__Bacteroidales	unclassified_o__Bacteroidales	OTU923
Proteobacteria	Enterobacteriaceae	Klebsiella	Klebsiella_pneumoniae_subsp._pneumoniae_g__Klebsiella	OTU896
Firmicutes	Lachnospiraceae	[Ruminococcus]_gnavus_group	uncultured_bacterium_g__[Ruminococcus]_gnavus_group	OTU892
Firmicutes	Lachnospiraceae	Robinsoniella	Robinsoniella_peoriensis_g__Robinsoniella	OTU914
Bacteroidetes	unclassified_o__Bacteroidales	unclassified_o__Bacteroidales	unclassified_o__Bacteroidales	OTU487
Bacteroidetes	Bacteroidaceae	Bacteroides	unclassified_g__Bacteroides	OTU855
Bacteroidetes	Bacteroidaceae	Bacteroides	unclassified_g__Bacteroides	OTU857
Firmicutes	Lachnospiraceae	Anaerostipes	Anaerostipes_caccae	OTU748
Bacteroidetes	Muribaculaceae	norank_f__Muribaculaceae	uncultured_bacterium_g__norank_f__Muribaculaceae	OTU852
Proteobacteria	norank_o__Rhodospirillales	norank_f__norank_o__Rhodospirillales	gut_metagenome_g__norank_f__norank_o__Rhodospirillales	OTU895
Bacteroidetes	Bacteroidaceae	Bacteroides	Bacteroides_caecimuris	OTU802
Bacteroidetes	Tannerellaceae	Parabacteroides	unclassified_g__Parabacteroides	OTU758
Firmicutes	Ruminococcaceae	Flavonifractor	unclassified_g__Flavonifractor	OTU766
Bacteroidetes	Bacteroidaceae	Bacteroides	unclassified_g__Bacteroides	OTU768
Firmicutes	Lachnospiraceae	Hungatella	uncultured_organism_g__Hungatella	OTU844
Bacteroidetes	unclassified_o__Bacteroidales	unclassified_o__Bacteroidales	unclassified_o__Bacteroidales	OTU948
Firmicutes	Erysipelotrichaceae	Coprobacillus	Coprobacillus_cateniformis	OTU738
Bacteroidetes	Bacteroidaceae	Bacteroides	Bacteroides_acidifaciens	OTU744
Verrucomicrobia	Akkermansiaceae	Akkermansia	unclassified_g__Akkermansia	OTU691
Bacteroidetes	unclassified_o__Bacteroidales	unclassified_o__Bacteroidales	unclassified_o__Bacteroidales	OTU908
Firmicutes	Peptostreptococcaceae	Clostridioides	Clostridioides_difficile_g__Clostridioides	OTU877
Firmicutes	Lachnospiraceae	Lachnoclostridium	unclassified_g__Lachnoclostridium	OTU876
Bacteroidetes	Muribaculaceae	norank_f__Muribaculaceae	unclassified_g__norank_f__Muribaculaceae	OTU915
Bacteroidetes	Muribaculaceae	norank_f__Muribaculaceae	unclassified_g__norank_f__Muribaculaceae	OTU849
Verrucomicrobia	Akkermansiaceae	Akkermansia	Akkermansia_muciniphila	OTU890
