# Supplementary material for: Shenlian (SL) Decoction, a Traditional Chinese Medicine Compound, May Ameliorate Blood Glucose via Mediating the Gut Microbiota in db/db Mice
Source: J Diabetes Res. 2022 Feb 9;2022:7802107. doi: 10.1155/2022/7802107 (PMC8855168; doi:10.1155/2022/7802107)
Supplement: Supplementary 2 — Supplementary Table 2: species classification information of 204 OTUs. [file 7802107.f2.pdf]

Phylum	Family	Genus	Species	OTU
Bacteroidetes	Muribaculaceae	norank_f__Muribaculaceae	uncultured_bacterium_g__norank_f__Muribaculaceae	OTU386
Bacteroidetes	Rikenellaceae	Alistipes	uncultured_bacterium_g__Alistipes	OTU432
Actinobacteria	Bifidobacteriaceae	Bifidobacterium	unclassified_g__Bifidobacterium	OTU395
Firmicutes	Lachnospiraceae	Lachnospiraceae_UCG-001	uncultured_bacterium_g__Lachnospiraceae_UCG-001	OTU531
Firmicutes	Streptococcaceae	Streptococcus	unclassified_g__Streptococcus	OTU761
Bacteroidetes	Muribaculaceae	norank_f__Muribaculaceae	uncultured_bacterium_g__norank_f__Muribaculaceae	OTU17
Bacteroidetes	Muribaculaceae	norank_f__Muribaculaceae	uncultured_bacterium_g__norank_f__Muribaculaceae	OTU18
Bacteroidetes	unclassified_o__Bacteroidales	unclassified_o__Bacteroidales	unclassified_o__Bacteroidales	OTU99
Bacteroidetes	Muribaculaceae	norank_f__Muribaculaceae	uncultured_bacterium_g__norank_f__Muribaculaceae	OTU1
Firmicutes	Ruminococcaceae	unclassified_f__Ruminococcaceae	unclassified_f__Ruminococcaceae	OTU458
Bacteroidetes	Muribaculaceae	norank_f__Muribaculaceae	uncultured_bacterium_g__norank_f__Muribaculaceae	OTU507
Bacteroidetes	Muribaculaceae	norank_f__Muribaculaceae	uncultured_bacterium_g__norank_f__Muribaculaceae	OTU136
Bacteroidetes	Muribaculaceae	norank_f__Muribaculaceae	uncultured_bacterium_g__norank_f__Muribaculaceae	OTU133
Firmicutes	Christensenellaceae	Christensenellaceae_R-7_group	bacterium_YE57	OTU202
Firmicutes	Ruminococcaceae	Anaerotruncus	uncultured_bacterium_g__Anaerotruncus	OTU333
Bacteroidetes	Prevotellaceae	Prevotellaceae_UCG-001	uncultured_Bacteroidales_bacterium_g__Prevotellaceae_UCG-001	OTU628
Bacteroidetes	Muribaculaceae	norank_f__Muribaculaceae	uncultured_bacterium_g__norank_f__Muribaculaceae	OTU181
Bacteroidetes	Muribaculaceae	norank_f__Muribaculaceae	uncultured_bacterium_g__norank_f__Muribaculaceae	OTU598
Bacteroidetes	Muribaculaceae	norank_f__Muribaculaceae	uncultured_bacterium_g__norank_f__Muribaculaceae	OTU182
Bacteroidetes	Muribaculaceae	norank_f__Muribaculaceae	uncultured_bacterium_g__norank_f__Muribaculaceae	OTU346
Bacteroidetes	Muribaculaceae	norank_f__Muribaculaceae	uncultured_bacterium_g__norank_f__Muribaculaceae	OTU613
Bacteroidetes	Bacteroidaceae	Bacteroides	unclassified_g__Bacteroides	OTU343
Firmicutes	Ruminococcaceae	unclassified_f__Ruminococcaceae	unclassified_f__Ruminococcaceae	OTU83
Bacteroidetes	Muribaculaceae	norank_f__Muribaculaceae	uncultured_bacterium_g__norank_f__Muribaculaceae	OTU813
Firmicutes	Ruminococcaceae	Butyricicoccus	uncultured_bacterium_g__Butyricicoccus	OTU342
Bacteroidetes	Muribaculaceae	norank_f__Muribaculaceae	uncultured_Bacteroidales_bacterium_g__norank_f__Muribaculaceae	OTU535
Firmicutes	Ruminococcaceae	Ruminiclostridium_9	uncultured_bacterium_g__Ruminiclostridium_9	OTU391
Bacteroidetes	Prevotellaceae	unclassified_f__Prevotellaceae	unclassified_f__Prevotellaceae	OTU142
Firmicutes	Ruminococcaceae	Oscillibacter	uncultured_bacterium_g__Oscillibacter	OTU317
Bacteroidetes	Muribaculaceae	norank_f__Muribaculaceae	uncultured_bacterium_g__norank_f__Muribaculaceae	OTU468
Bacteroidetes	Prevotellaceae	Prevotellaceae_UCG-001	uncultured_Bacteroidales_bacterium_g__Prevotellaceae_UCG-001	OTU715
Firmicutes	Ruminococcaceae	GCA-900066225	unclassified_g__GCA-900066225	OTU412
Bacteroidetes	Rikenellaceae	Alistipes	uncultured_bacterium_g__Alistipes	OTU541
Bacteroidetes	Muribaculaceae	norank_f__Muribaculaceae	uncultured_bacterium_g__norank_f__Muribaculaceae	OTU80
Bacteroidetes	Muribaculaceae	norank_f__Muribaculaceae	uncultured_bacterium_g__norank_f__Muribaculaceae	OTU87
Firmicutes	Lachnospiraceae	norank_f__Lachnospiraceae	unclassified_g__norank_f__Lachnospiraceae	OTU500
Bacteroidetes	Muribaculaceae	norank_f__Muribaculaceae	uncultured_bacterium_g__norank_f__Muribaculaceae	OTU462

Bacteroidetes	Muribaculaceae	norank_f__Muribaculaceae	uncultured_bacterium_g__norank_f__Muribaculaceae	OTU461
Bacteroidetes	Muribaculaceae	norank_f__Muribaculaceae	uncultured_bacterium_g__norank_f__Muribaculaceae	OTU464
Bacteroidetes	Muribaculaceae	norank_f__Muribaculaceae	unclassified_g__norank_f__Muribaculaceae	OTU475
Firmicutes	Lachnospiraceae	Lachnoclostridium	unclassified_g__Lachnoclostridium	OTU313
Firmicutes	Ruminococcaceae	Ruminiclostridium_5	Ruminiclostridium_sp._KB18	OTU416
Bacteroidetes	Muribaculaceae	norank_f__Muribaculaceae	uncultured_bacterium_g__norank_f__Muribaculaceae	OTU388
Bacteroidetes	Muribaculaceae	norank_f__Muribaculaceae	uncultured_bacterium_g__norank_f__Muribaculaceae	OTU796
Firmicutes	Lachnospiraceae	norank_f__Lachnospiraceae	Clostridium_sp._Culture-27	OTU380
Firmicutes	Lachnospiraceae	unclassified_f__Lachnospiraceae	unclassified_f__Lachnospiraceae	OTU70
Firmicutes	Lachnospiraceae	Lachnospiraceae_NK4A136_group	uncultured_bacterium_g__Lachnospiraceae_NK4A136_group	OTU172
Firmicutes	Ruminococcaceae	[Eubacterium]_coprostanoligenes_group	unclassified_g__[Eubacterium]_coprostanoligenes_group	OTU430
Actinobacteria	Eggerthellaceae	Enterorhabdus	uncultured_bacterium_g__Enterorhabdus	OTU78
Bacteroidetes	Muribaculaceae	norank_f__Muribaculaceae	uncultured_bacterium_g__norank_f__Muribaculaceae	OTU607
Bacteroidetes	Muribaculaceae	norank_f__Muribaculaceae	uncultured_bacterium_g__norank_f__Muribaculaceae	OTU353
Bacteroidetes	unclassified_o__Bacteroidales	unclassified_o__Bacteroidales	unclassified_o__Bacteroidales	OTU596
Firmicutes	Ruminococcaceae	Oscillibacter	uncultured_bacterium_g__Oscillibacter	OTU293
Firmicutes	Lachnospiraceae	unclassified_f__Lachnospiraceae	unclassified_f__Lachnospiraceae	OTU396
Actinobacteria	Eggerthellaceae	Enterorhabdus	uncultured_bacterium_g__Enterorhabdus	OTU124
Bacteroidetes	Rikenellaceae	Alistipes	unclassified_g__Alistipes	OTU231
Firmicutes	Lachnospiraceae	norank_f__Lachnospiraceae	unclassified_g__norank_f__Lachnospiraceae	OTU441
Firmicutes	Ruminococcaceae	Ruminococcaceae_UCG-014	uncultured_rumen_bacterium_g__Ruminococcaceae_UCG-014	OTU51
Bacteroidetes	unclassified_o__Bacteroidales	unclassified_o__Bacteroidales	unclassified_o__Bacteroidales	OTU720
Firmicutes	Lachnospiraceae	norank_f__Lachnospiraceae	unclassified_g__norank_f__Lachnospiraceae	OTU453
Firmicutes	Lactobacillaceae	Lactobacillus	uncultured_bacterium_g__Lactobacillus	OTU303
Firmicutes	Ruminococcaceae	Ruminococcus_1	unclassified_g__Ruminococcus_1	OTU319
Firmicutes	Erysipelotrichaceae	Turicibacter	uncultured_bacterium_g__Turicibacter	OTU6
Firmicutes	Ruminococcaceae	Oscillibacter	[Clostridium]_leptum_g__Oscillibacter	OTU283
Bacteroidetes	Muribaculaceae	norank_f__Muribaculaceae	uncultured_bacterium_g__norank_f__Muribaculaceae	OTU420
Firmicutes	Erysipelotrichaceae	Erysipelatoclostridium	uncultured_bacterium_g__Erysipelatoclostridium	OTU27
Bacteroidetes	Muribaculaceae	norank_f__Muribaculaceae	uncultured_bacterium_g__norank_f__Muribaculaceae	OTU429
Bacteroidetes	Muribaculaceae	norank_f__Muribaculaceae	uncultured_Bacteroidales_bacterium_g__norank_f__Muribaculaceae	OTU4
Firmicutes	Ruminococcaceae	Ruminococcaceae_UCG-010	uncultured_organism_g__Ruminococcaceae_UCG-010	OTU263
Bacteroidetes	Muribaculaceae	norank_f__Muribaculaceae	uncultured_Bacteroidales_bacterium_g__norank_f__Muribaculaceae	OTU9
Firmicutes	Clostridiales_vadinBB60_group	norank_f__Clostridiales_vadinBB60_group	unclassified_g__norank_f__Clostridiales_vadinBB60_group	OTU212
Bacteroidetes	Muribaculaceae	norank_f__Muribaculaceae	uncultured_bacterium_g__norank_f__Muribaculaceae	OTU102
Bacteroidetes	Muribaculaceae	norank_f__Muribaculaceae	uncultured_bacterium_g__norank_f__Muribaculaceae	OTU109
Bacteroidetes	Muribaculaceae	norank_f__Muribaculaceae	uncultured_bacterium_g__norank_f__Muribaculaceae	OTU108
Firmicutes	Clostridiales_vadinBB60_group	norank_f__Clostridiales_vadinBB60_group	uncultured_bacterium_g__norank_f__Clostridiales_vadinBB60_group	OTU187

Firmicutes	Peptococcaceae	norank_f__Peptococcaceae	unclassified_g__norank_f__Peptococcaceae	OTU638
Firmicutes	Lachnospiraceae	Lachnospiraceae_NK4A136_group	unclassified_g__Lachnospiraceae_NK4A136_group	OTU281
Bacteroidetes	Muribaculaceae	norank_f__Muribaculaceae	uncultured_bacterium_g__norank_f__Muribaculaceae	OTU197
Bacteroidetes	Muribaculaceae	norank_f__Muribaculaceae	uncultured_bacterium_g__norank_f__Muribaculaceae	OTU190
Bacteroidetes	Muribaculaceae	norank_f__Muribaculaceae	uncultured_bacterium_g__norank_f__Muribaculaceae	OTU150
Bacteroidetes	Muribaculaceae	Muribaculum	uncultured_bacterium_g__Muribaculum	OTU557
Bacteroidetes	Rikenellaceae	Alistipes	uncultured_bacterium_g__Alistipes	OTU602
Bacteroidetes	Muribaculaceae	norank_f__Muribaculaceae	unclassified_g__norank_f__Muribaculaceae	OTU556
Bacteroidetes	Muribaculaceae	norank_f__Muribaculaceae	uncultured_bacterium_g__norank_f__Muribaculaceae	OTU639
Epsilonbacteraeot	Helicobacteraceae	Helicobacter	unclassified_g__Helicobacter	OTU747
Firmicutes	Lactobacillaceae	Lactobacillus	unclassified_g__Lactobacillus	OTU177
Firmicutes	Lachnospiraceae	[Eubacterium]_xylanophilum_group	uncultured_bacterium_g__[Eubacterium]_xylanophilum_group	OTU393
Bacteroidetes	unclassified_o__Bacteroidales	unclassified_o__Bacteroidales	unclassified_o__Bacteroidales	OTU25
Firmicutes	Lachnospiraceae	Lachnospiraceae_NK4A136_group	uncultured_bacterium_g__Lachnospiraceae_NK4A136_group	OTU334
Firmicutes	Peptococcaceae	norank_f__Peptococcaceae	unclassified_g__norank_f__Peptococcaceae	OTU354
Bacteroidetes	Muribaculaceae	norank_f__Muribaculaceae	uncultured_bacterium_g__norank_f__Muribaculaceae	OTU804
Cyanobacteria	norank_o__Gastranaerophilales	norank_f__norank_o__Gastranaerophilales	uncultured_bacterium_g__norank_f__norank_o__Gastranaerophilales	OTU728
Bacteroidetes	Tannerellaceae	Parabacteroides	unclassified_g__Parabacteroides	OTU305
Firmicutes	Lachnospiraceae	Blautia	unclassified_g__Blautia	OTU297
Firmicutes	Lachnospiraceae	ASF356	uncultured_bacterium_g__ASF356	OTU642
Deferribacteres	Deferribacteraceae	Mucispirillum	uncultured_bacterium_g__Mucispirillum	OTU881
Firmicutes	Lachnospiraceae	Lachnospiraceae_NK4A136_group	uncultured_Clostridiales_bacterium_g__Lachnospiraceae_NK4A136_group	OTU201
Firmicutes	Erysipelotrichaceae	norank_f__Erysipelotrichaceae	unclassified_g__norank_f__Erysipelotrichaceae	OTU636
Bacteroidetes	Muribaculaceae	norank_f__Muribaculaceae	uncultured_bacterium_g__norank_f__Muribaculaceae	OTU443
Firmicutes	Ruminococcaceae	Ruminiclostridium_6	uncultured_bacterium_g__Ruminiclostridium_6	OTU445
Bacteroidetes	Tannerellaceae	Parabacteroides	unclassified_g__Parabacteroides	OTU35
Bacteroidetes	Muribaculaceae	norank_f__Muribaculaceae	uncultured_bacterium_g__norank_f__Muribaculaceae	OTU560
Actinobacteria	Eggerthellaceae	Enterorhabdus	uncultured_bacterium_g__Enterorhabdus	OTU447
Bacteroidetes	Prevotellaceae	Prevotellaceae_UCG-001	uncultured_Bacteroidales_bacterium_g__Prevotellaceae_UCG-001	OTU608
Bacteroidetes	unclassified_o__Bacteroidales	unclassified_o__Bacteroidales	unclassified_o__Bacteroidales	OTU455
Firmicutes	Ruminococcaceae	Anaerotruncus	uncultured_bacterium_g__Anaerotruncus	OTU449
Bacteroidetes	Muribaculaceae	norank_f__Muribaculaceae	uncultured_Bacteroidales_bacterium_g__norank_f__Muribaculaceae	OTU43
Bacteroidetes	Muribaculaceae	norank_f__Muribaculaceae	uncultured_bacterium_g__norank_f__Muribaculaceae	OTU206
Bacteroidetes	Prevotellaceae	unclassified_f__Prevotellaceae	unclassified_f__Prevotellaceae	OTU57
Proteobacteria	Burkholderiaceae	Oligella	Oligella_ureolytica	OTU97
Bacteroidetes	Rikenellaceae	Rikenella	uncultured_bacterium_g__Rikenella	OTU603
Firmicutes	Lachnospiraceae	unclassified_f__Lachnospiraceae	unclassified_f__Lachnospiraceae	OTU349
Bacteroidetes	Muribaculaceae	norank_f__Muribaculaceae	uncultured_bacterium_g__norank_f__Muribaculaceae	OTU42

Bacteroidetes	Muribaculaceae	norank_f__Muribaculaceae	uncultured_bacterium_g__norank_f__Muribaculaceae	OTU41
Bacteroidetes	Tannerellaceae	Parabacteroides	unclassified_g__Parabacteroides	OTU132
Bacteroidetes	Marinifilaceae	Odoribacter	unclassified_g__Odoribacter	OTU637
Bacteroidetes	Muribaculaceae	norank_f__Muribaculaceae	uncultured_bacterium_g__norank_f__Muribaculaceae	OTU39
Firmicutes	Erysipelotrichaceae	norank_f__Erysipelotrichaceae	unclassified_g__norank_f__Erysipelotrichaceae	OTU436
Firmicutes	Ruminococcaceae	Ruminococcaceae_UCG-014	unclassified_g__Ruminococcaceae_UCG-014	OTU382
Bacteroidetes	Muribaculaceae	norank_f__Muribaculaceae	uncultured_bacterium_g__norank_f__Muribaculaceae	OTU401
Firmicutes	Clostridiales_vadinBB60_group	norank_f__Clostridiales_vadinBB60_group	unclassified_g__norank_f__Clostridiales_vadinBB60_group	OTU271
Actinobacteria	Eggerthellaceae	unclassified_f__Eggerthellaceae	unclassified_f__Eggerthellaceae	OTU141
Firmicutes	Ruminococcaceae	Ruminococcaceae_UCG-013	unclassified_g__Ruminococcaceae_UCG-013	OTU463
Firmicutes	Lachnospiraceae	norank_f__Lachnospiraceae	Clostridium_sp._Culture-54	OTU241
Bacteroidetes	Prevotellaceae	unclassified_f__Prevotellaceae	unclassified_f__Prevotellaceae	OTU159
Patescibacteria	Saccharimonadaceae	Candidatus_Saccharimonas	uncultured_bacterium_g__Candidatus_Saccharimonas	OTU446
Firmicutes	Ruminococcaceae	Ruminococcaceae_UCG-010	unclassified_g__Ruminococcaceae_UCG-010	OTU440
Bacteroidetes	Muribaculaceae	norank_f__Muribaculaceae	unclassified_g__norank_f__Muribaculaceae	OTU411
Firmicutes	Lachnospiraceae	Lachnospiraceae_UCG-006	unclassified_g__Lachnospiraceae_UCG-006	OTU705
Bacteroidetes	Muribaculaceae	norank_f__Muribaculaceae	unclassified_g__norank_f__Muribaculaceae	OTU56
Firmicutes	Lachnospiraceae	Tyzzerella	uncultured_bacterium_g__Tyzzerella	OTU312
Firmicutes	Clostridiales_vadinBB60_group	norank_f__Clostridiales_vadinBB60_group	uncultured_bacterium_g__norank_f__Clostridiales_vadinBB60_group	OTU243
Bacteroidetes	Muribaculaceae	norank_f__Muribaculaceae	uncultured_bacterium_g__norank_f__Muribaculaceae	OTU48
Actinobacteria	Eggerthellaceae	DNF00809	uncultured_bacterium_g__DNF00809	OTU710
Bacteroidetes	Muribaculaceae	norank_f__Muribaculaceae	uncultured_bacterium_g__norank_f__Muribaculaceae	OTU621
Firmicutes	Lachnospiraceae	unclassified_f__Lachnospiraceae	unclassified_f__Lachnospiraceae	OTU615
Firmicutes	Lachnospiraceae	unclassified_f__Lachnospiraceae	unclassified_f__Lachnospiraceae	OTU612
Bacteroidetes	Muribaculaceae	norank_f__Muribaculaceae	uncultured_bacterium_g__norank_f__Muribaculaceae	OTU582
Firmicutes	Lachnospiraceae	norank_f__Lachnospiraceae	Clostridium_sp._Clone-17	OTU473
Firmicutes	Lachnospiraceae	norank_f__Lachnospiraceae	unclassified_g__norank_f__Lachnospiraceae	OTU456
Firmicutes	Lachnospiraceae	norank_f__Lachnospiraceae	unclassified_g__norank_f__Lachnospiraceae	OTU459
Bacteroidetes	Bacteroidaceae	Bacteroides	unclassified_g__Bacteroides	OTU559
Firmicutes	Lachnospiraceae	Lachnoclostridium	unclassified_g__Lachnoclostridium	OTU315
Bacteroidetes	Muribaculaceae	norank_f__Muribaculaceae	uncultured_Barnesiella_sp._g__norank	OTU113
Bacteroidetes	Muribaculaceae	norank_f__Muribaculaceae	uncultured_bacterium_g__norank_f__Muribaculaceae	OTU71
Actinobacteria	Eggerthellaceae	Enterorhabdus	unclassified_g__Enterorhabdus	OTU330
Bacteroidetes	Muribaculaceae	norank_f__Muribaculaceae	uncultured_bacterium_g__norank_f__Muribaculaceae	OTU537
Bacteroidetes	Muribaculaceae	norank_f__Muribaculaceae	uncultured_bacterium_g__norank_f__Muribaculaceae	OTU28
Firmicutes	Ruminococcaceae	norank_f__Ruminococcaceae	unclassified_g__norank_f__Ruminococcaceae	OTU299
Bacteroidetes	Muribaculaceae	norank_f__Muribaculaceae	unclassified_g__norank_f__Muribaculaceae	OTU593
Bacteroidetes	Muribaculaceae	norank_f__Muribaculaceae	uncultured_bacterium_g__norank_f__Muribaculaceae	OTU96

Bacteroidetes	Muribaculaceae	norank_f__Muribaculaceae	uncultured_bacterium_g__norank_f__Muribaculaceae	OTU90
Proteobacteria	Moraxellaceae	Psychrobacter	unclassified_g__Psychrobacter	OTU687
Actinobacteria	Eggerthellaceae	Enterorhabdus	uncultured_bacterium_g__Enterorhabdus	OTU451
Bacteroidetes	unclassified_o__Bacteroidales	unclassified_o__Bacteroidales	unclassified_o__Bacteroidales	OTU467
Firmicutes	Ruminococcaceae	Ruminococcaceae_UCG-010	unclassified_g__Ruminococcaceae_UCG-010	OTU409
Firmicutes	Ruminococcaceae	Ruminococcaceae_UCG-005	uncultured_bacterium_g__Ruminococcaceae_UCG-005	OTU344
Firmicutes	Ruminococcaceae	Ruminiclostridium_6	uncultured_bacterium_g__Ruminiclostridium_6	OTU474
Firmicutes	Ruminococcaceae	Ruminiclostridium_9	unclassified_g__Ruminiclostridium_9	OTU833
Bacteroidetes	Rikenellaceae	Alistipes	uncultured_bacterium_g__Alistipes	OTU491
Bacteroidetes	Muribaculaceae	norank_f__Muribaculaceae	uncultured_bacterium_g__norank_f__Muribaculaceae	OTU331
Firmicutes	Clostridiaceae_1	Candidatus_Arthromitus	Candidatus_Arthromitus_sp._SFB-mouse-Japan	OTU545
Firmicutes	Ruminococcaceae	unclassified_f__Ruminococcaceae	unclassified_f__Ruminococcaceae	OTU650
Firmicutes	Ruminococcaceae	unclassified_f__Ruminococcaceae	unclassified_f__Ruminococcaceae	OTU651
Bacteroidetes	Muribaculaceae	norank_f__Muribaculaceae	unclassified_g__norank_f__Muribaculaceae	OTU69
Bacteroidetes	Rikenellaceae	Alistipes	unclassified_g__Alistipes	OTU19
Firmicutes	Lachnospiraceae	unclassified_f__Lachnospiraceae	unclassified_f__Lachnospiraceae	OTU657
Bacteroidetes	Marinifilaceae	Odoribacter	unclassified_g__Odoribacter	OTU431
Bacteroidetes	Rikenellaceae	Alistipes	uncultured_bacterium_g__Alistipes	OTU584
Bacteroidetes	Prevotellaceae	unclassified_f__Prevotellaceae	unclassified_f__Prevotellaceae	OTU435
Firmicutes	Ruminococcaceae	norank_f__Ruminococcaceae	unclassified_g__norank_f__Ruminococcaceae	OTU581
Firmicutes	Lachnospiraceae	Roseburia	unclassified_g__Roseburia	OTU439
Firmicutes	Lachnospiraceae	norank_f__Lachnospiraceae	uncultured_bacterium_g__norank_f__Lachnospiraceae	OTU712
Firmicutes	Christensenellaceae	Christensenellaceae_R-7_group	uncultured_rumen_bacterium_g__Christensenellaceae_R-7_group	OTU199
Proteobacteria	Desulfovibrionaceae	norank_f__Desulfovibrionaceae	uncultured_Desulfovibrionales_bacterium_g__norank_f__Desulfovibrionaceae	OTU216
Firmicutes	Lachnospiraceae	Lachnospiraceae_UCG-001	uncultured_bacterium_g__Lachnospiraceae_UCG-001	OTU508
Firmicutes	Lachnospiraceae	GCA-900066575	uncultured_bacterium_g__GCA-900066575	OTU384
Firmicutes	Lachnospiraceae	norank_f__Lachnospiraceae	uncultured_bacterium_g__norank_f__Lachnospiraceae	OTU713
Bacteroidetes	Muribaculaceae	norank_f__Muribaculaceae	uncultured_bacterium_g__norank_f__Muribaculaceae	OTU550
Bacteroidetes	Prevotellaceae	Alloprevotella	gut_metagenome_g__Alloprevotella	OTU356
Firmicutes	Ruminococcaceae	Ruminococcaceae_NK4A214_group	uncultured_bacterium_g__Ruminococcaceae_NK4A214_group	OTU359
Firmicutes	Ruminococcaceae	Ruminiclostridium	uncultured_bacterium_g__Ruminiclostridium	OTU321
Bacteroidetes	Rikenellaceae	Rikenellaceae_RC9_gut_group	unclassified_g__Rikenellaceae_RC9_gut_group	OTU36
Bacteroidetes	Muribaculaceae	norank_f__Muribaculaceae	uncultured_bacterium_g__norank_f__Muribaculaceae	OTU433
Firmicutes	Lachnospiraceae	Blautia	Blautia_coccoides_g__Blautia	OTU268
Firmicutes	Lachnospiraceae	Lachnospiraceae_NK4A136_group	unclassified_g__Lachnospiraceae_NK4A136_group	OTU714
Firmicutes	Lachnospiraceae	Blautia	unclassified_g__Blautia	OTU327
Firmicutes	Ruminococcaceae	Ruminococcaceae_NK4A214_group	unclassified_g__Ruminococcaceae_NK4A214_group	OTU114
Firmicutes	Ruminococcaceae	Ruminococcaceae_UCG-013	unclassified_g__Ruminococcaceae_UCG-013	OTU250

Bacteroidetes	Marinifilaceae	Odoribacter	uncultured_bacterium_g__Odoribacter	OTU554
Bacteroidetes	Muribaculaceae	norank_f__Muribaculaceae	uncultured_bacterium_g__norank_f__Muribaculaceae	OTU273
Firmicutes	Clostridiales_vadinBB60_group	norank_f__Clostridiales_vadinBB60_group	unclassified_g__norank_f__Clostridiales_vadinBB60_group	OTU644
Bacteroidetes	Muribaculaceae	norank_f__Muribaculaceae	uncultured_bacterium_g__norank_f__Muribaculaceae	OTU786
Bacteroidetes	Rikenellaceae	Rikenellaceae_RC9_gut_group	unclassified_g__Rikenellaceae_RC9_gut_group	OTU383
Firmicutes	Ruminococcaceae	Ruminiclostridium	unclassified_g__Ruminiclostridium	OTU488
Bacteroidetes	Muribaculaceae	norank_f__Muribaculaceae	uncultured_bacterium_g__norank_f__Muribaculaceae	OTU655
Firmicutes	Lachnospiraceae	norank_f__Lachnospiraceae	uncultured_bacterium_g__norank_f__Lachnospiraceae	OTU387
Firmicutes	Erysipelotrichaceae	norank_f__Erysipelotrichaceae	uncultured_bacterium_g__norank_f__Erysipelotrichaceae	OTU652
Bacteroidetes	Rikenellaceae	Rikenellaceae_RC9_gut_group	uncultured_bacterium_g__Rikenellaceae_RC9_gut_group	OTU771
Firmicutes	Lachnospiraceae	unclassified_f__Lachnospiraceae	unclassified_f__Lachnospiraceae	OTU437
Firmicutes	Ruminococcaceae	Ruminiclostridium_9	uncultured_bacterium_g__Ruminiclostridium_9	OTU122
Firmicutes	Clostridiales_vadinBB60_group	norank_f__Clostridiales_vadinBB60_group	unclassified_g__norank_f__Clostridiales_vadinBB60_group	OTU936
Firmicutes	Ruminococcaceae	Ruminococcaceae_UCG-014	unclassified_g__Ruminococcaceae_UCG-014	OTU282
Bacteroidetes	Prevotellaceae	Prevotellaceae_UCG-001	uncultured_Bacteroidales_bacterium_g__Prevotellaceae_UCG-001	OTU193
